# How do caregivers know when to take their child for immunizations?

**DOI:** 10.1186/1471-2431-5-44

**Published:** 2005-11-29

**Authors:** Kate M Shaw, Lawrence E Barker

**Affiliations:** 1 National Immunization Program Centers for Disease Control and Prevention Atlanta, Georgia

## Abstract

**Background:**

Childhood vaccinations help reduce and eliminate many causes of morbidity and mortality among children. The objective of this study was to compare 4:3:1:3:3 (4+ doses of diphtheria and tetanus toxoids and pertussis vaccine, 3+ doses of poliovirus vaccine, 1+ doses of measles-containing vaccine, 3+ doses of *Haemophilus influenzae *type b vaccine, and 3+ doses of hepatitis B vaccine) coverage among children whose caregivers learned by different methods when their child's most recent immunization was needed.

**Methods:**

Between July 2001 and December 2002, a portion of households receiving the National Immunization Survey were asked how they knew when to take the child in for his/her most recent immunization. Responses were post-coded into several categories: 'Doctor/nurse reminder at previous immunization visit', 'Shot card/record', 'Reminder/recall', and 'Other'. Respondents could give more than one answer. Children who did not receive any vaccines, had ≤ 1 visits for vaccinations, or whose caregiver did not provide an answer to the question were excluded from analyses. Chi-square analyses were used to compare 4:3:1:3:3 coverage among 19–35 month old children.

**Results:**

Children whose caregivers indicated that a doctor/nurse told them at a previous immunization visit when to return for the next immunization had significantly greater 4:3:1:3:3 coverage than those who did not choose the response (77.2% vs. 70.1%, p < 0.01). However, no significant difference in coverage was found between households that did/did not indicate that reminder/recalls (71.0% vs. 75.5%, p = 0.24) helped them remember when to take their child for their most recent immunization visit; only borderline significance was found between those that did/did not choose shot cards (70.6% vs. 76.2%, p = 0.07).

**Conclusion:**

A doctor or nurse's reminder during an immunization visit of the next scheduled immunization visit effectively encourages caregivers to bring children in for immunizations, providing an inexpensive and easy way to effectively increase immunization coverage.

## Background

Caregivers learn when their children need immunizations in a variety of ways. For example, providers can remind caregivers, during an immunization visit, of the next scheduled immunization visit, or use reminder/recall systems. Less formally, caregivers can learn of needed immunization visits through relatives or friends, through daycare requirements, or other methods. To assess how the way caregivers know when their child needs immunizations impacts 4:3:1:3:3 (4 or more doses of diphtheria and tetanus toxoids and pertussis vaccine, 3 or more doses of poliovirus vaccine, 1 or more doses of measles-containing vaccine, 3 or more doses of *Haemophilus influenzae *type b vaccine, and 3 or more doses of hepatitis B vaccine) coverage, we examined data from a question administered to a random subsample of the households in the National Immunization Survey (NIS).

## Methods

The NIS is a random-digit-dialing survey conducted annually by the Centers for Disease Control and Prevention to obtain vaccination coverage for the U.S. non-institutionalized population of children aged 19 to 35 months. To obtain vaccination information, a follow-up survey is mailed to all of the eligible children's immunization health providers [1]. The data were weighted to represent the sampling design, number of land-line telephones per house, provider response propensity, and a number of other factors. This makes the results nationally representative. Details about the design and weighting have been previously published [2,3].

Between July 2001 and December 2002, a random sample of 9,908 (from the 49,385 NIS respondents) were asked the open-ended question, "How did you know when to take your child for his/her most recent immunization?". We post-coded responses into four categories: (1) doctor/nurse told me at a previous immunization visit when to come back for the next shot, (2) shot card/record had schedule, (3) reminder/recalls (which included responses like: outreach worker called/came to house to tell me; health department called me/sent me reminder; and physician's office called me/sent me reminder), and (4) other (which included responses like: relative/friend told me; found out during visit to doctor or other health care provider; day care/headstart requirement; WIC nurse told me; government program requirement; and other infrequent responses). Respondents could give more than one answer. Children who did not receive any vaccines, had ≤ 1 visits for vaccinations, or whose caregiver did not provide an answer to the question were excluded from analyses. Sufficient data from providers was obtained from 7,810 (78.8%) of the respondents. Of those 7,687 (98.4%) were used in the analyses, including determination of 4:3:1:3:3 immunization status.

We used chi-square analyses to test for associations between methods of learning when the child needs immunizations (both overall and stratified by demographics) and immunization status. We conducted a stepwise logistic regression analysis to identify associations between the responses chosen and immunization, controlling for demographics. Candidate factors for the stepwise regression were: did/did not rely on each of the methods listed to determine when the child's last immunization was needed and child's race/ethnicity and birth order and maternal marital status, education level, age, poverty status, residence in a Metropolitan Statistical Area (MSA), time from last immunization to interview, and child's age at interview. Time from last immunization to interview and child's age at interview were added to the model to control for the effect of recall bias.

All estimates and standard errors were calculated using SAS release 8.02 (SAS, Cary, NC, 1999) and SAS-callable SUDAAN release 8.0.0 (RTI, RTP, NC, 2001), a software package designed to analyze complex survey data [4]. We conducted all statistical tests with two-tailed alternatives, α = 0.05.

## Results

Results for the question "How did you know when to take your child for his/her most recent immunization?" appear in Table 1. Since respondents could give multiple answers, percents add to more than 100. Table 1 summarizes frequencies of responses and 4:3:1:3:3 coverage for those who did/did not choose each response and difference of these coverages. Coverage for those who responded 'doctor/nurse told at previous immunization visit' was significantly greater than that for those who did not so respond (77.2% vs. 70.1%, p < 0.01). No other significant differences were found (shot card, p = 0.07; reminder/recall, p = 0.24; other p = 0.86).

**Table 1:** How Caregivers Know When to Take Their Child for Immunizations and UTD 4:3:1:3:3^a ^Coverage, United States, National Immunization Survey, Parental Knowledge and Experiences Questionnaire, July 2001 – December 2002.^b^

**How did you know when to take your child for his/her most recent immunization?**	**ChoseResponse^c ^% (95% CI)^d^**	**UTD4:3:1:3:3 Chose Response % (95% CI)**	**UTD4:3:1:3:3 Did Not Chose Response % (95% CI)**	**UTD 4:3:1:3:3 Difference % points (95% CI)**
Doctor/Nurse at Previous Immunization Visit ^e^	70.3 (68.3, 72.3)	77.2 (74.9, 79.5)	70.1 (66.0, 74.1)	7.1 (2.5, 11.8)
Shot Card/Record	19.5 (17.6, 21.3)	70.6 (65.2, 76.0)	76.2 (74.0, 78.3)	-5.6 (-11.3, 0.2)
Reminder/Recall^f^	8.6 (7.4, 9.8)	71.0 (63.8, 78.1)	75.5 (73.4, 77.6)	-4.5 (-11.9, 2.9)
Other^g^	18.3 (16.7, 19.9)	75.4 (71.4, 79.5)	75.0 (72.7, 77.3)	0.4 (-4.3, 5.1)

a 4 or more doses of diphtheria and tetanus toxoids and pertussis vaccine, 3 or more doses of poliovirus vaccine, 1 or more doses of measles-containing vaccine, 3 or more doses of *Haemophilus influenzae *type b vaccine, and 3 or more doses of hepatitis B vaccine at 19–35 months of age

b Children in the Q3/2001–Q4/2002 National Immunization Survey were born between August 1998 and June 2001.

c Respondents could choose more than one response. Thus, total % of those who chose response will not add to 100%.

d Percents are weighted estimates.

e p-value < 0.01 for chi-square test comparing % UTD 4:3:1:3:3 who chose the reason and % UTD 4:3:1:3:3 who did not choose the reason

f Outreach worker called/came to house to tell me, health department called me/sent me reminder, or physician's office called me/sent me reminder

g Relative/friend told me, found out during visit to doctor or other healthcare provider, day care/headstart requirement, WIC (Women, Infants, Children) nurse told me, government program requirement, or other

The stepwise logistic regression (Table 2) retained the factor 'doctor/nurse at previous immunization visit' (adjusted odds ratio (AOR): 1.4, 95% CI: 1.0–1.8) and the demographics: child's birth order, maternal age, poverty status, MSA, time from last immunization to interview, and child's age at interview. Other ways of learning when to take a child for the most recent immunization visit were forced to remain in the model, but were not found to be significantly associated with immunization (shot card: AOR: 0.9, 95% CI: 0.7–1.2; reminder/recall: AOR: 1.1, 95% CI: 0.7–1.7; other: AOR: 1.2, 95% CI: 0.8–1.6). Because time from last immunization to interview and age at interview were significant factors, there appears to be some recall bias. However, since 'doctor/nurse at previous immunization visit' remained significantly associated with immunization after controlling from time and age we can conclude that recall bias and age are not the driving factors.

**Table 2:** How Caregivers Know When to Take Their Child for Immunizations. Adjusted Odds Ratios Predicting Up-to-date Status for 4:3:1:3:3 ^a^, United States, National Immunization Survey, Parental Knowledge and Experiences Questionnaire, July 2001 – December 2002.^b^

	**Variable**	**Adjusted Odds Ratio**	**95% CI**
How did you know when to take your child for his/her most recent immunization?	Doctor/Nurse at Previous Immunization Visit		
	Chosen ^c^	1.4	(1.0, 1.8) ^d^
	Not Chosen	1.0	Referent
	Shot Card/Record		
	Chosen	0.9	(0.7, 1.2)
	Not Chosen	1.0	Referent
	Reminder/Recall ^e^		
	Chosen	1.1	(0.7, 1.7)
	Not Chosen	1.0	Referent
	Other ^f^		
	Chosen	1.2	(0.8, 1.6)
	Not Chosen	1.0	Referent

	First Born Status		
	No	1.0	Referent
	Yes	1.5	(1.2, 1.8)
	Age of Mother		
	≤ 19 Years	1.0	Referent
	20–29 Years	0.8	(0.4, 1.4)
	≥ 30 Years	1.2	(0.6, 2.2)
	Poverty Status ^g^		
	Above, > $75 K	2.0	(1.4, 2.9)
	Above, ≤ $75 K	1.6	(1.2, 2.1)
	Below	1.0	Referent
	Unknown	2.2	(1.5, 3.4)
	Metropolitan Statistical Area (MSA) Status		
	MSA Central City	1.0	Referent
	MSA Non-Central City	1.4	(1.1, 1.7)
	Non-MSA	1.2	(0.9, 1.7)
	Time from Last Immunization to Interview		
	≤ 6 months	2.8	(1.9, 4.1)
	7 – 12 months	2.0	(1.5, 2.7)
	≥ 13 months	1.0	Referent
	Age of Child at Interview		
	19 – 24 months	0.3	(0.2, 0.4)
	25 – 29 months	0.6	(0.5, 0.8)
	30 – 35 months	1.0	Referent

a 4 or more doses of diphtheria and tetanus toxoids and pertussis vaccine, 3 or more doses of poliovirus vaccine, 1 or more doses of measles-containing vaccine, 3 or more doses of *Haemophilus influenzae *type b vaccine, and 3 or more doses of hepatitis B vaccine at 19–35 months of age

b Children in the Q3/2001–Q4/2002 National Immunization Survey were born between August 1998 and June 2001.

c Respondents could choose more than one response

d The lower bound was 1.01; the confidence interval (1.01, 1.8) does not contain 1.0.

e Outreach worker called/came to house to tell me, health department called me/sent me reminder, or physician's office called me/sent me reminder

f Relative/friend told me, found out during visit to doctor or other healthcare provider, day care/headstart requirement, WIC (Women, Infants, and Children) nurse told me, government program requirement, or other

g Poverty level depends on household income, year data was collected, and number of people living in household and is determined by the US Bureau of Census poverty threshold.

Table 3 displays relationships between demographics and method of learning when a child needed the most recent immunization. Several characteristics were significantly associated with responses. Among respondents with a household income below the federal poverty level, 12.0% (95% CI: 9.0–15.1%) chose 'reminder/recall', while 8.3% or fewer of the respondents in the other income categories did (income >$75 K: 6.4%, 95% CI: 4.2–8.6 %; above poverty level, income ≤ $75 K: 8.3%, 95% CI: 6.6–9.9%; unknown income: 6.8%, 95% CI: 3.4–10.2%). Of respondents who had a MSA status of 'non-MSA', 14.0% (95% CI: 10.9–17.1 %) chose 'reminder/recall', while 7.5% or fewer respondents in the other MSA status categories did (MSA central city: 7.1%, 95% CI: 5.6–8.6%; MSA non-central city: 7.5%, 95% CI: 5.6–9.4%). Mothers of age over 30 years (73.0%, 95%CI: 70.3–75.6%) and married mothers (72.5%, 95% CI:70.2–74.8%) were more likely to rely on a doctor/nurse at a previous immunization visit. We found no significant associations between response and child's race/ethnicity, time from last immunization to interview, and child's age at interview.

**Table 3:** How Caregivers Know When to Take Their Child for Immunizations and Demographic Characteristics of Respondents, United States, National Immunization Survey, Parental Knowledge and Experiences Questionnaire, July 2001 – December 2002.^a^

**Characteristic (%) ^b^**	**Doctor/Nurse ^c ^% (95% CI) ^d^**	**Shot Card/Record % (95% CI)**	**Reminder/Recall ^e ^% (95% CI)**	**Other ^f ^% (95% CI)**
Child's Race/Ethnicity				
P-value	0.08	0.16	0.38	0.46
Hispanic (23.8)	69.4 (65.0, 73.8)	17.5 (13.9, 21.1)	6.9 (4.5, 9.2)	20.5 (16.4, 24.6)
Non-Hispanic, White (56.7)	71.7 (69.2, 74.3)	19.8 (17.3, 22.3)	8.9 (7.3, 10.5)	17.8 (15.9, 19.7)
Non-Hispanic, Black (14.4)	64.6 (58.8, 70.4)	22.7 (17.7, 27.7)	10.1 (6.8, 13.4)	17.8 (13.4, 22.2)
Non-Hispanic, Other (5.1)	74.8 (68.6, 80.9)	15.4 (10.5, 20.2)	9.0 (5.4, 12.6)	15.1 (9.6, 20.5)
Marital Status of Mother				
P-value	0.01	0.06	0.50	0.07
Widowed/Divorced/Separated/Deceased (8.4)	64.9 (57.7, 72.1)	14.5 (10.5, 18.6)	7.7 (3.9, 11.4)	26.0 (19.0, 33.0)
Never Married (20.4)	65.0 (60.2, 69.7)	21.7 (17.1, 26.4)	9.8 (7.5, 12.2)	18.7 (14.9, 22.5)
Married (71.1)	72.5 (70.2, 74.8)	19.4 (17.2, 21.5)	8.3 (6.9, 9.8)	17.3 (15.5, 19.0)
Education Level of Mother				
P-value	0.01	0.05	<0.01	0.08
<12 Years (16.3)	71.2 (66.1, 76.3)	20.1 (14.8, 25.4)	7.5 (5.1, 9.8)	14.1 (10.1, 18.1)
12 Years (36.0)	66.2 (62.4, 70.1)	20.6 (17.1, 24.1)	11.9 (9.3, 14.5)	17.9 (15.1, 20.8)
>12 Years, Non College Graduate (14.3)	69.4 (64.6, 74.2)	22.7 (18.3, 27.1)	7.1 (5.0, 9.1)	21.3 (17.0, 25.6)
College Graduate (33.4)	74.7 (71.9, 77.4)	16.5 (14.2, 18.8)	6.2 (4.6, 7.8)	19.4 (16.9, 22.0)
First Born Status				
P-value	<0.01	0.05	0.71	0.14
No (62.8)	67.3 (64.6, 70.0)	20.8 (18.4, 23.3)	8.7 (7.2, 10.3)	19.2 (17.1, 21.3)
Yes (37.2)	75.4 (72.7, 78.1)	17.1 (14.4, 19.9)	8.3 (6.6, 10.0)	16.8 (14.3, 19.2)
Age of Mother				
P-value	0.03	0.39	<0.01	0.34
≤ 19 Years (3.5)	68.3 (57.7, 79.0)	25.3 (15.1, 35.6)	13.0 (5.5, 20.5)	12.4 (4.2, 20.6)
20–29 Years (45.6)	67.5 (64.3, 70.7)	20.0 (16.9, 23.0)	10.8 (8.8, 12.8)	18.1 (15.7, 20.6)
≥ 30 Years (50.9)	73.0 (70.3, 75.6)	18.6 (16.3, 20.9)	6.3 (4.9, 7.7)	18.8 (16.6, 21.1)
Poverty Status				
P-value	<0.01	0.02	0.03	0.65
Above, > $75 K (16.7)	74.6 (70.7, 78.4)	14.7 (11.9, 17.5)	6.4 (4.2, 8.6)	19.7 (16.2, 23.2)
Above, ≤ $75 K (50.7)	71.7 (69.0, 74.4)	20.5 (17.9, 23.1)	8.3 (6.6, 9.9)	18.2 (16.1, 20.4)
Below (21.2)	61.8 (56.7, 67.0)	20.1 (15.9, 24.2)	12.0 (9.0, 15.1)	18.7 (14.8, 22.7)
Unknown (11.5)	73.7 (67.4, 79.9)	20.6 (13.7, 27.4)	6.8 (3.4, 10.2)	15.6 (10.3, 20.9)
MSA Status				
P-value	<0.01	0.19	<0.01	0.22
MSA Central City (34.4)	69.1 (65.8, 72.4)	20.1 (17.3, 22.9)	7.1 (5.6, 8.6)	19.6 (16.7, 22.6)
MSA Non-Central City (47.2)	75.2 (72.4, 77.9)	17.8 (15.0, 20.5)	7.5 (5.6, 9.4)	16.8 (14.4, 19.1)
Non-MSA (18.5)	60.1 (55.1, 65.1)	22.7 (17.9, 27.5)	14.0 (10.9, 17.1)	19.6 (16.2, 23.0)
Time from Last Immunization to Interview				
P-value	0.38	0.25	0.80	0.51
19–24 months (39.4)	70.6 (67.4, 73.7)	17.7 (15.1, 20.2)	8.6 (6.8, 10.4)	18.9 (16.3, 21.5)
25–29 months (34.0)	71.8 (68.6, 75.0)	20.0 (16.8, 23.1)	8.1 (6.1, 10.1)	17.0 (14.4, 19.6)
30–35 months (26.6)	68.0 (63.8, 72.3)	21.5 (17.4, 25.5)	9.2 (6.7, 11.7)	19.0 (15.8, 22.3)
Age of Child at Interview				
P-value	0.69	0.86	0.28	0.37
19–24 months (36.4)	71.1 (67.8, 74.3)	18.8 (16.1, 21.6)	9.1 (7.1, 11.1)	17.0 (14.4, 19.5)
25–29 months (29.4)	69.1 (65.6, 72.5)	19.6 (16.5, 22.7)	9.5 (7.2, 11.7)	18.2 (15.3, 21.1)
30–35 months (34.3)	70.6 (67.0, 74.1)	20.0 (16.4, 23.6)	7.3 (5.3, 9.3)	19.8 (16.8, 22.7)

a Children in the Q3/2001–Q4/2002 National Immunization Survey were born between August 1998 and June 2001.

b Weight frequencies of respondents.

c Respondents could choose more than one response. Thus, total % of those who chose response will not add to 100%.

d Percents are weighted estimates.

e Outreach worker called/came to house to tell me, health department called me/sent me reminder, or physician's office called me/sent me reminder

f Relative/friend told me, found out during visit to doctor or other healthcare provider, day care/headstart requirement, WIC (Women, Infants, Children) nurse told me government program requirement, or other

## Discussion

We found no difference in immunization coverage among those who did/did not indicate that 'reminder/recall' told respondents when to take children in for the most recent immunization. Similarly, we found no difference among those who said a shot card did/did not indicate when a child needed immunizations (although, at the α = 0.10 level, we would have found a negative association between relying on shot cards and immunization status). In comparison, households indicating that a doctor or nurse told them on a previous visit when the child needed immunization had significantly greater coverage.

Demographics differed among those who indicated various methods of knowing when to take a child in for the most recent immunization visit. However, when we controlled for demographics through a logistic regression model, 'doctor/nurse at previous immunization visit' remained significant. The other ways of knowing when to take the child for the most recent immunization visit were not significant.

The relative paucity of respondents indicating 'reminder/recall' (8.5%) could indicate that providers are not widely using reminder/recall, that caregivers do not rely on it for knowing when children need immunization, or that caregivers tend to forget reminder/recalls. The data do not allow us to choose among these explanations. However, other studies have shown that reminder/recalls are not widely used by physicians. In 1992, of the 1175 pediatricians and family physicians surveyed by Szilagyi et al., only 13% used computer or reminder files to identify undervaccinated children [5]. Nine years later, similar results were found by Tierney et al. Among 433 pediatricians surveyed, only 16% were currently using routine reminder or recall messages [6].

Despite the low use of reminder/recalls, they have long featured in ways to increase immunization coverage. In 1993, the National Vaccine Advisory Committee (NVAC) recommended that all public and private health-care providers operate a tracking system which generates reminders and recalls, and issued another such recommendation in 1999 [7,8] Again in 1998, the Advisory Committee on Immunization Practices, the American Academy of Pediatrics, and the American Academy of Family Physicians jointly recommended the use of a reminder and/or recall system by vaccination providers [9]. Many published studies have found that reminder/recall increase vaccination coverage [10-15].

## Conclusion

Although reminder/recalls are an effective method of increasing immunization coverage, our results suggest that taking time at each immunization visit to remind caregivers about the next immunization visit might also be powerful. Verbal reminders given while caregivers are present are cheap, easy, and possibly quite effective. Our study did not find a positive association between reminder/recalls and immunization coverage. However, several other studies have shown that provider recommendation is significantly associated with increase in immunization coverage [16-21]. Our data do not provide sufficient detail to explain this discrepancy.

This study has several limitations. First, methods of determining when the child needed the most recent immunization were determined from caregivers' memories. This possibly introduced recall bias, particularly if ability to recall use of a particular method and immunization are both associated with some unobserved factor. Second, all children in the survey were at least 19 months of age, and the question was only asked about the 'most recent' immunization. The bulk of immunizations are scheduled for the first year of life. It is possible that the 'most recent' immunization is not typical of earlier immunizations. Third, NIS relies on provider-verified immunization history; coverage could be underestimated due to incomplete records and reporting. This study is also subject to other limitations such as the inability to determine the sole impact of the provider on immunization coverage. Doctors/nurses who effectively use verbal reminders may also be more effective at immunizing their patients, and so, higher immunization coverage may not result solely from the verbal reminder. Finally, the dichotomization of immunization coverage does not take into account the varying levels of completeness for the 4:3:1:3:3 series.

## Competing interests

The author(s) declare that they have no competing interests.

## Authors' contributions

KS carried out the analysis and interpretation of the data and the drafting of the manuscript. LB participated in revisions of the manuscript and provided statistical expertise and supervision. Both authors read and approved the final manuscript.

## Pre-publication history

The pre-publication history for this paper can be accessed here:


